# Dual Role of Reactive Oxygen Species and their Application in Cancer Therapy

**DOI:** 10.7150/jca.54699

**Published:** 2021-07-25

**Authors:** Run Huang, Huan Chen, Jiayu Liang, Yi Li, Jiali Yang, Chuang Luo, Youshan Tang, Yu Ding, Xing Liu, Qing Yuan, Hong Yu, Yingchun Ye, Wenfeng Xu, Xiang Xie

**Affiliations:** Public Center of Experimental Technology, The school of Basic Medical Sciences, Southwest Medical University, Luzhou, Sichuan Province, 646000, China.

**Keywords:** reactive oxygen species, cancer, pro-tumor, anti-cancer, self-adaption system, therapy

## Abstract

Reactive oxygen species (ROS) play a dual role in the initiation, development, suppression, and treatment of cancer. Excess ROS can induce nuclear DNA, leading to cancer initiation. Not only that, but ROS also inhibit T cells and natural killer cells and promote the recruitment and M2 polarization of macrophages; consequently, cancer cells escape immune surveillance and immune defense. Furthermore, ROS promote tumor invasion and metastasis by triggering epithelial-mesenchymal transition in tumor cells. Interestingly, massive accumulation of ROS inhibits tumor growth in two ways: (1) by blocking cancer cell proliferation by suppressing the proliferation signaling pathway, cell cycle, and the biosynthesis of nucleotides and ATP and (2) by inducing cancer cell death via activating endoplasmic reticulum stress-, mitochondrial-, and P53- apoptotic pathways and the ferroptosis pathway. Unfortunately, cancer cells can adapt to ROS via a self-adaption system. This review highlighted the bidirectional regulation of ROS in cancer. The study further discussed the application of massively accumulated ROS in cancer treatment. Of note, the dual role of ROS in cancer and the self-adaptive ability of cancer cells should be taken into consideration for cancer prevention.

## Introduction

The Global Burden of Disease 2017 Study showed that deaths from noncommunicable diseases represented 73.4% of deaths in 2017 1. Cancer causes the second largest number of fatalities following cardiovascular disease 1. Between 2007 and 2017, the number of cancer cases increased by 33%, and the average annual age-standardized incidence rates for all cancers increased 2. Global cancer statistics 2018 revealed 18.1 million new cancer cases and 9.6 million cancer-related deaths in 2018 3. Malignant tumors have become the main killer among humans. The treatment of cancer has gained the attention of the medical community because of the rapid increase in the global cancer burden. An increasing number of studies have emerged demonstrating that reactive oxygen species (ROS) are highly intertwined with various cancers 4, such as lung cancer 5, colorectal cancer 6, breast cancer 7, hepatocellular cancer 8, and cervical cancer 9, which are among the top 10 cancers ranked by the highest number of incident cases globally 2. The mechanism of ROS in carcinogenesis needs to be considered to determine more efficient therapeutics against cancer. This review highlighted the 'double-edged sword' role of ROS in cancer formation, development, and suppression. In addition, the self-adaption mechanism of cancer cells was discussed. The study also focused on the therapeutic approaches targeting ROS to treat cancers, thus providing an insight into how pro- and anti-tumorigenic ROS signaling pathways are involved in the treatment of cancer.

## Physical properties of ROS

ROS exist in two forms. One form comprises free oxygen radicals including superoxide, hydroxyl radicals, nitric oxide, alkoxyl radicals, and peroxyl radicals 10. The other form comprises nonradical ROS, including hydrogen peroxide (H_2_O_2_), organic hydroperoxides, and hypochlorite 10. ROS are mainly generated by the mitochondrial electron transport chain and tricarboxylic acid cycle 11. Besides, the peroxisome and the endoplasmic reticulum (ER) can produce oxidants 12. Many enzymes also promote the production of ROS, such as nicotinamide adenine dinucleotide phosphate oxidase (NOX), xanthine oxidase, nitric oxide synthase, cyclooxygenases, cytochrome P450 enzymes, and lipoxygenases 12. Additionally, exogenous stress, such as that from ionizing radiation, chemotherapeutic drugs and environmental insult, is also the main cause of ROS generation 13. ROS can oxidize intracellular lipids, proteins and deoxyribonucleic acid (DNA), thus leading to the accumulation of damaged biological molecules. ROS induced DNA damage can include single- or double-strand breakage, base modifications, deoxyribose modification, and DNA cross-linking 13. Moreover, lipid peroxidation results in the formation of reactive aldehydes, including malondialdehyde and 4-hydroxy-2-nonenal, which have high reactivity toward protein and DNA 14. Although this damaging aspect of ROS biology persists, oxidant species are increasingly being understood to have beneficial effects. ROS can regulate cellular proliferation, differentiation, survival, and even apoptosis processes 15. ROS thus play a dual role in cell development.

## Potential mechanisms involved in the cancer promoting effects of ROS

### Promoting effects of ROS in cancer

The physiological levels of ROS are important to regulate crucial cellular processes such as cellular proliferation, differentiation, survival, and even apoptosis in normal cells 15. Once ROS exceed normal levels, the high levels of ROS can initiate tumorigenesis and promote tumor progression 16. Excess ROS cause gene mutations, and subsequently activate oncogenes and inhibit tumor suppressor genes, such as Kirsten rat sarcoma viral oncogene (KRAS) and tumor suppressor P53, thus leading to cancer initiation 17. Not only that, but increased ROS also promote tumor development by reducing the function of T cells and natural killer (NK) cells and promoting the recruitment and M2 polarization of macrophages, thereby promoting tumor progression 18-21. Infiltration of T cells, NK cells, and M1 macrophages in breast cancer has been reported to prevent tumor development 22. Furthermore, high levels of ROS induce tumor invasion and metastasis via epithelial-mesenchymal transition (EMT) 23. Below, we further discuss the potential mechanisms of ROS in promoting cancer.

### Potential mechanisms of promoting effects of ROS in cancer

#### ROS promote cancer formation via initiating DNA mutation

DNA is the carrier of genetic information essential for the survival and reproduction of organisms. The evolution of normal cells into cancer cells involves genetic instability 24. The overexpression of exogenous and endogenous ROS often leads to DNA mutations, affecting the interpretation and transmission of genetic information 25. The DNA mutations lead to the inactivation of tumor suppressor genes and/or the activation of oncogenes, allowing cells to proliferate outside their normal growth restraints, and promote tumorigenesis by favoring tumor cell proliferation, migration, and resistance to apoptosis 26. ROS can cause hotspot codon mutagenesis in P53, and the mutations of P53 have been reported in liver cancer and breast cancer 25, 27. Besides, ROS also cause mutagenesis in the oncogene rat sarcoma (*Ras*) gene, and the mutation of *Ras* is closely related to skin cancer and colorectal cancer 28-30. In another study, excessive H_2_O_2_ produced by myeloid cells has been shown to trigger genome-wide DNA mutations in intestinal epithelial cells to stimulate invasive growth during intestinal tumorigenesis 16.

**Fig 1:**
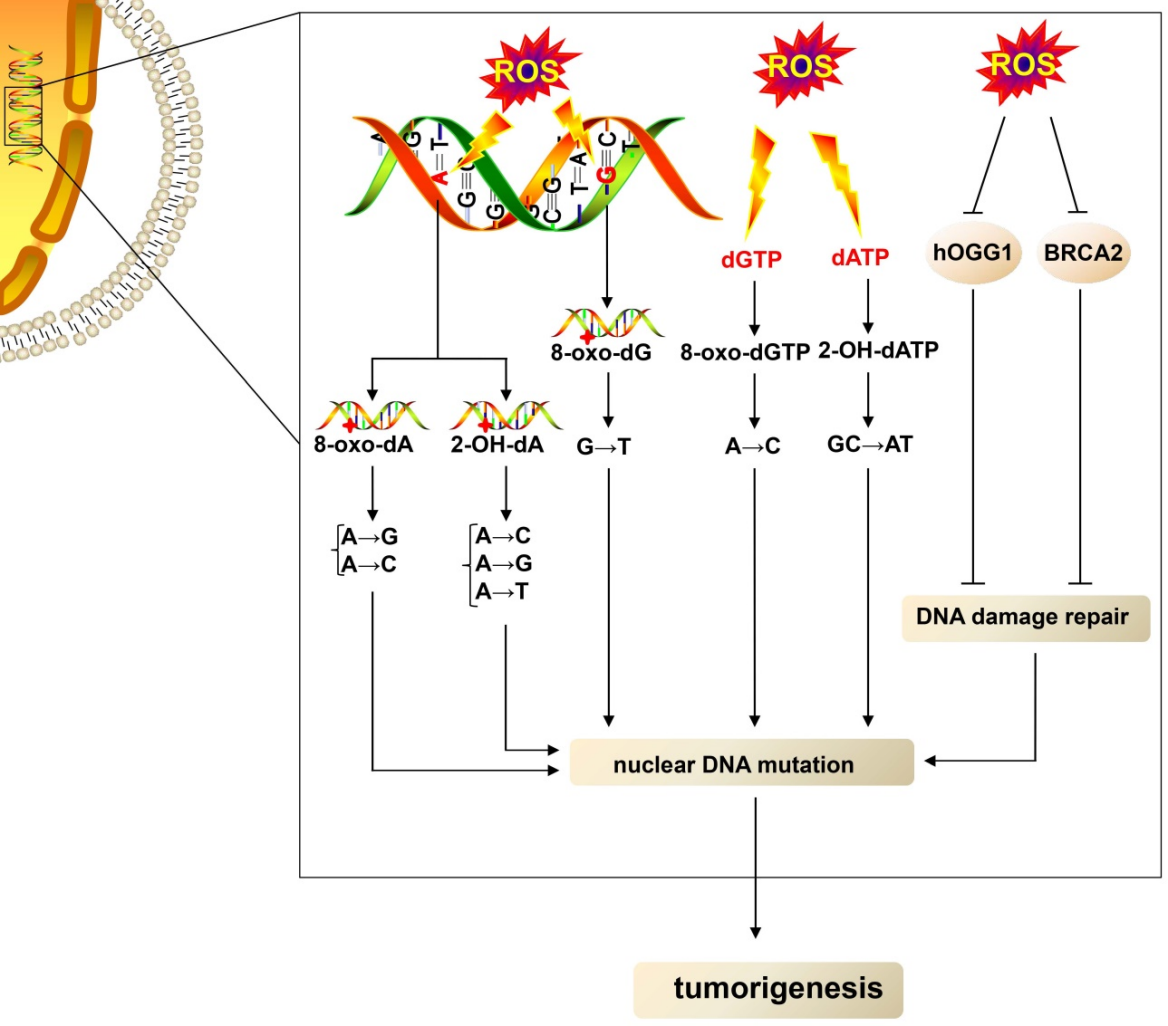
** Elevated-ROS cause nuclear DNA, thus leading to tumorigenesis.** High levels of ROS oxidize adenine in DNA, thus leading to the formation of 8-oxo-dA and 2-OH-dA; in turn, 8-oxo-dA can lead to A→G and A→C mutations, and 2-OH-dA causes A→C, A→G, and A→T substitutions. ROS also oxidize guanine in DNA, resulting in the formation of 8-oxo-dG, which leads G→T transversion mutations. dGTP and dATP in the nucleotide pool are also oxidized by ROS, yielding 8-oxo-dGTP and 2-OH-dATP. The misincorporation of 8-oxo-dGTP and 2-OH-dATP as a substrate causes A→C and GC→AT mutation, respectively. Moreover, ROS impair the DNA damage repair system by downregulating the function of hOGG1 and BRCA2, thus leading to nuclear DNA mutations and favoring tumorigenesis. dATP, deoxyadenosine triphosphate; BRCA2, breast cancer susceptibility gene 2.

ROS can lead to DNA damage and cause DNA mutations (Fig. 1). ROS can oxidize nucleobases and form different types of oxidative damage products that have mutational properties 31. Guanine (G), with a lower oxidation potential compared with other bases 32, is most easily oxidized by ROS, leading to the formation of an oxidized product 7,8-dihydro-8-oxo-2´-deoxyguanosine (8-oxo-dG), which is the most extensively studied oxidative damage product 33. 8-oxo-dG acts as a mutagenic lesion due to the loss of base-pairing specificity and mispairing with adenine (A) 34. When ROS oxidizes guanine in DNA, 8-oxo-dG acts as a template, leading to G→T (thymine) transversion mutations 35. When ROS oxidize deoxyguanosine triphosphate in the nucleotide pool, 8-oxo-dG acts as a substrate, resulting in A→C (cytosine) mutations 35. ROS also oxidize A with two main products: 2-hydroxy-2'-deoxyadenosine (2-OH-dA) 36 and 7,8-dihydro-8-oxo-2'-deoxyadenosine (8-oxo-dA) 32. Mutagenic replication of 2-OH-dA as a template causes A→C, A→G, A→T substitutions, and misincorporation of 2-OH-dA as a substrate causes GC→AT transition 36, 37. Besides, 8-oxo-dA can lead to A→G and A→C mutations 38. Other oxidative DNA lesions, such as 5-hydroxy-2'-deoxycytidine, 5,6-dihydro-5,6-dihydroxy-2'-deoxyuridine, and 5-hydroxy-2'-deoxyuridine oxidized from cytidine, as well as 5,6-dihydro-5,6-dihydroxy-2'-deoxythymidine oxidized from thymine, have also been shown to be mutagenic 31. In addition, ROS can cause hydrogen bond breakage, unfolding, and double- and single-strand breakage of the DNA double-helix structure, leading to the exposure of more purine and pyrimidine residues to ROS that further facilitate the oxidation of nucleobases 39, which promotes DNA mutations.

The DNA damage repair system is vital in dealing with DNA damage to avoid DNA mutations**.** DNA damage repair methods include base excision repair (BER) for base modifications, mismatch repair for mismatched bases, nucleotide excision repair (NER) for intra-strand cross-links and thymidine dimers, and nonhomologous end joining and homologous recombination (HR) for double-strand DNA breaks 40. ROS can not only damage DNA but also repress the part DNA repair process (Fig. 1). ROS can oxidize human 8-oxoguanine DNA N-glycosylase 1 (hOGG1) and inhibit its activity, resulting in the impairment of BER 41. Besides, ROS block HR by inducing proteasomal degradation of breast cancer susceptibility gene 2, one of the key factors for HR 42. In summary, ROS not only cause direct DNA damage but also impair the DNA damage repair system, leading to DNA mutations and thus favoring tumor growth.

#### ROS promote cancer development via regulating immune cells

##### T cells

**Fig 2:**
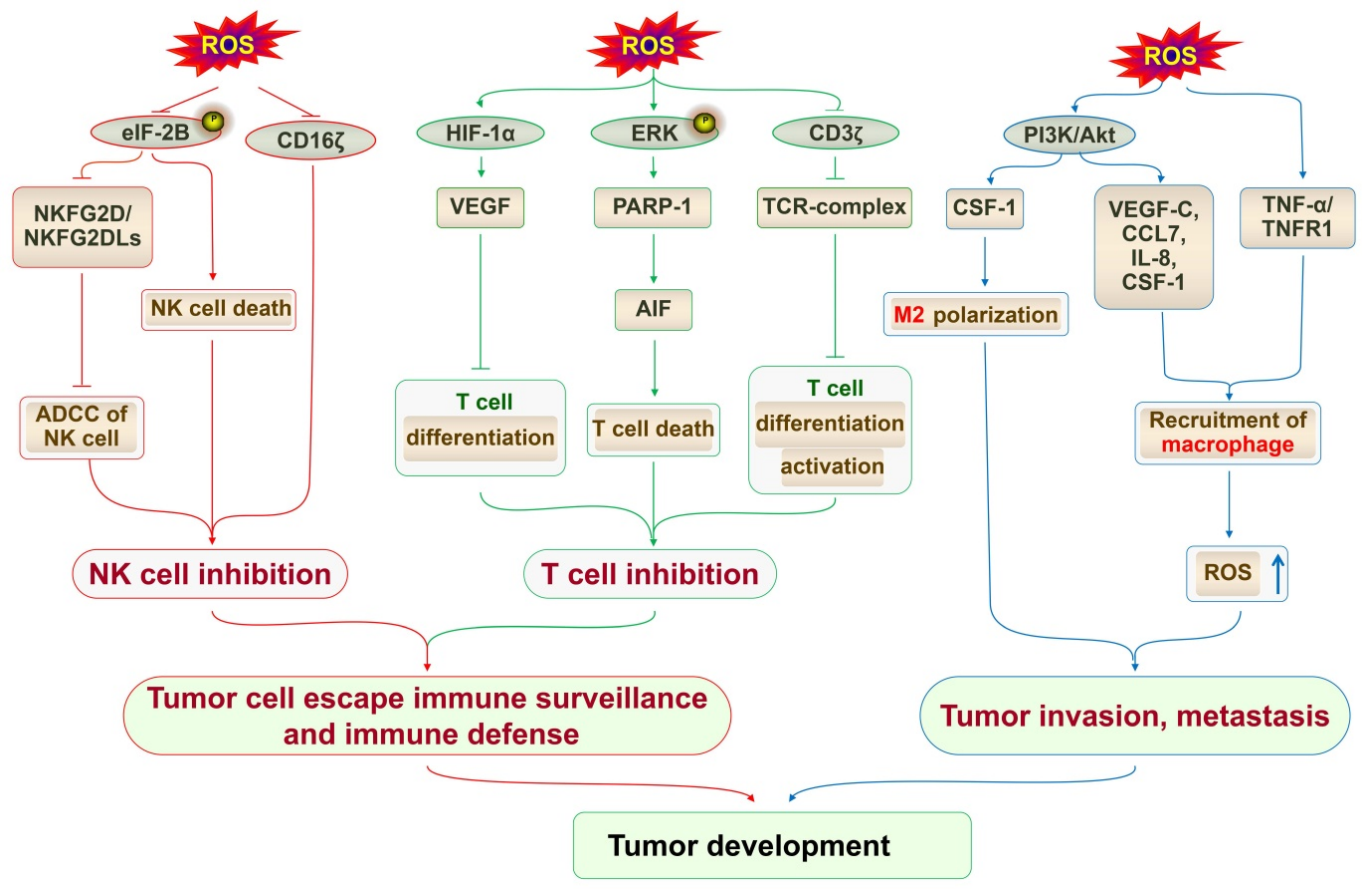
** The upregulation of ROS inhibits T cells and NK cells, recruits macrophages, and induces M2 macrophage polarization.** The overproduction of ROS attenuates the phosphorylation of eIF2B, thereby downregulating NKG2D/NKG2Dls and suppressing NK cell-mediated ADCC activity. Elevated ROS downregulate CD16ζ expression in NK cells, thus inhibiting the cytotoxicity of NK cells. The upregulation of ROS enhances the production of VEGF via stabilizing HIF-1α, thus interfering with T-cell differentiation. ROS trigger ERK activation of PARP-1, thereby causing the release of AIF and finally leading to T-cell death. The production of ROS also decreases CD3ζ chain expression, thereby impairing the formation of TCR, and blocking the differentiation and activation of T cells. The inhibition of T cells and NK cells favors tumor development. Increased ROS activate the PI3K/Akt signaling pathway and promote the secretion of VEGF-C, CCL7, IL-8, and CSF-1, thus resulting in macrophage recruitment. ROS also activate TNF-α/TNFR1 signaling, which results in macrophage recruitment. Macrophages in turn elevate the secretion of ROS and finally promote tumor invasion and metastasis. ROS/PI3K/Akt signaling-dependent CSF-1 production induces M2 polarization of macrophages, thus contributing to tumor cell growth. NKG2DLs, NKG2D ligands; ADCC, antibody-dependent cell-mediated cytotoxicity; AIF, apoptosis-inducing factor; CCL7, cytokines chemokine (C-C motif) ligand 7.

High levels of ROS can block the differentiation, maturation, and activation of T cells and induce T-cell death to suppress the antitumor function of T cells (Fig. 2). The upregulation of ROS enhances the production of vascular endothelial growth factor (VEGF) via stabilizing hypoxia-inducible factor 1 α (HIF-1α) 43, 44. The overproduction of VEGF then blocks the differentiation and/or migration of thymus-committed progenitors, thus interfering with T-cell development and contributing to tumor-associated immune deficiencies 45. The production of ROS can also reduce the expression of the CD3ζ chain, thus blocking the differentiation and activation of T cells 46, 47. The CD3ζ chain, a component of the T-cell antigen receptor complex, is important in T-cell differentiation and activation, which mediates signal transduction of T cells 47. With the decrease in the expression of the CD3ζ chain, the functions of T cells, such as proliferative ability, cytotoxic activity, and cytokine production are inhibited, finally suppressing the immune response 46-48. In line with this, the reduced expression of CD3ζ has been reported in several cancers, such as gastric adenocarcinoma, breast cancer, and head and neck cancer 49-51. In the tumor microenvironment, granulocytic or polymorphonuclear myeloid-derived suppressor cells, which are immunosuppressive cells, have the ability to overexpress ROS to inhibit T-cell activation and function, thus promoting tumor metastasis 18. A reduction of ROS in myeloid-derived suppressor cells significantly increases the number of active CD8^+^ and CD4^+^ T lymphocytes, which efficiently suppresses the proliferation of tumor cells 52. Besides influencing the development of T cells, elevated levels of ROS also induce T-cell death. ROS trigger extracellular signal-regulated kinase (ERK) phosphorylation and subsequently lead to the activation of poly(ADP-ribose) polymerase (PARP) 1, which causes the release of apoptosis-inducing factor, leading to T-cell death 53. Consistent with these findings, the *in vivo* blockade of ROS reversed the selective loss of selective CD4+ T lymphocytes and delayed nonalcoholic fatty liver disease-promoted hepatocellular carcinoma (HCC) 54.

##### NK cells

Besides controlling T cells, high levels of ROS facilitate cancer cell distant metastasis by downregulating NK cell function (Fig. 2). Elevated levels of ROS downregulate CD16ζ expression in NK cells, which is an important signal transduction molecule for NK cell functions, thus inhibiting the cytotoxicity of NK cells 46. Besides, the overproduction of ROS can attenuate the phosphorylation of eukaryotic initiation factor 2B, downregulate the expression of natural killer group 2 member D (NKG2D) and NKG2D ligands, and subsequently reduce the release of cytotoxic granules of NK cells, thus suppressing NK cell-mediated antibody-dependent cell-mediated cytotoxicity activity and finally promoting breast cancer growth and metastasis 7, 55. Furthermore, ROS reduce interferon-γ (IFN-γ) production in NK cells, consequently downregulating NK cell-mediated clearance of malignant cells and finally resulting in melanoma metastasis 56. Elevated levels of ROS also can lead to the death of NK cells by inducing apoptosis 57. Primary human chronic myelomonocytic leukemia (CMML) cells release ROS, leading to the death of NK cells, which work as an immune escape mechanism in CMML 58. Additionally, liver-resident NK cells undergo apoptosis because of the increased production of mitochondrial ROS, which allows tumors to evade liver NK cell surveillance, ultimately leading to colorectal liver metastatic tumors 19.

##### Macrophages

Macrophages are of two types: cancer-inhibiting M1 type and cancer-promoting M2 type 59. Tumor-associated macrophages (TAMs) show an M2 phenotype, the infiltration of which contributes to cancer malignant progression 60. ROS can regulate both recruitment and M2 polarization of macrophages to promote cancer development 20 (Fig. 2). ROS have been reported to inactivate phosphatase and tensin homolog 61, a well-known negative regulator of phosphatidylinositide 3-kinase (PI3K), the activated form of which phosphorylates protein kinase B (Akt) 62. The mechanisms reported so far suggest that NOX4-induced ROS activate the PI3K/Akt signaling pathway and lead to the secretion of VEGF-C, cytokines chemokine (C-C motif) ligand 7, interleukin-8, and colony-stimulating factor-1 (CSF-1), resulting in macrophage recruitment 20. Besides, myeloid-derived H_2_O_2_ activates tumor necrosis factor (TNF-α)/TNFR1 signaling in intestinal epithelial cells (IEC), increasing the recruitment of macrophages and in turn elevating the secretion of H_2_O_2_, which can trigger IEC mutation and promote tumor invasion 16. In addition, ROS are essential to polarize macrophages toward the M2 status 21. A previous study revealed that long-term arsenic exposure could trigger M2 macrophage differentiation via ROS production 63. Studies have found that the reduction of ROS production specifically blocks the differentiation of M2 macrophages 21. ROS/PI3K/Akt signaling-dependent CSF-1 production induces M2 polarization of macrophages, thus contributing to tumor cell growth 20. Of note, the high levels of ROS promote TAMs to generate TNF-α in the primary melanoma microenvironment, thus promoting tumor invasion and metastasis 64.

#### ROS promote cancer invasion and metastasis via EMT

**Fig 3:**
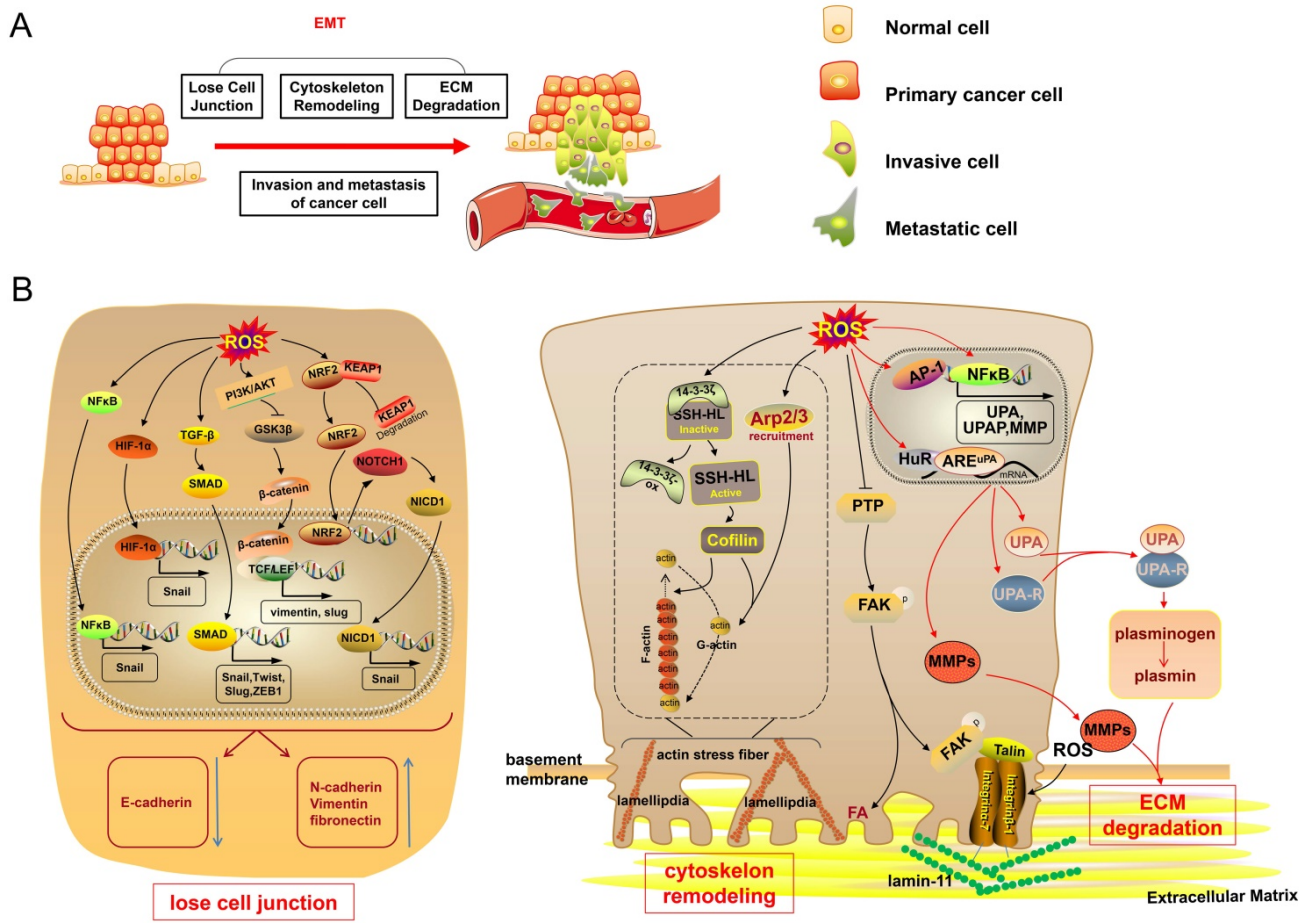
** Excess ROS trigger EMT, promoting cancer cell invasion and metastasis.** (**A**) When undergoing EMT, cancer cells experience cell-cell junction dissociation, cytoskeleton remodeling and ECM degradation. These processes of EMT endow cancer cells with invasive and metastatic capacities. (**B**) High levels of ROS promote the expression of the transcription factors Snail, Slug, Twist and ZEB1 via the NF-κB, HIF-1α, TGF-β/SMAD, PI3K/Akt/GSK-3β and NRF2/NOTHC1/NICD1 signaling pathways, thereby repressing the expression of E-cadherin and promoting N-cadherin, Vimentin, and fibronectin, disrupting cell-cell junctions and initiating the EMT process. Elevated-ROS oxidize 14-3-3ζ and subsequently activate SSH-1L, thus resulting in the activation of cellular cofilin depolymerizing F-actin. Increased-ROS also recruit Arp2/3. Cofilin and Arp2/3, thus resulting in maintenance of actin treadmilling, formation of lamellipodia and cytoskeletal extension. Increased ROS inhibit PTP, thereby sustaining the phosphorylation of FAK, and recruiting talin, which reacts with the extracellular domain of integrin; integrin α7β1 modified by ROS links to laminin-111 in the ECM. ROS promote AP-1 and NF-κB activation of the transcription of MMPs, uPA, and uPAR; ROS also enhance the binding of HuR with ARE^uPA^ and consequently promote uPA and uPAR expression. Activated uPA leads to the transformation of plasminogen into plasmin. MMPs and plasmin result in ECM degradation. NICD1, NOTHC1 intracellular domain; N-cadherin, neural cadherin; PTP, protein tyrosine phosphatase.

EMT is a process in which epithelial cells transform into mesenchymal cells. Upon the initiation of EMT, epithelial cells lose cell-cell junctions and cell polarity. Then, the cells experience both cytoskeleton remodeling and extracellular matrix (ECM) protein degradation 65. These processes of EMT have endowed cancer cells with invasive and metastatic capacities. Finally, the cancer cells undergoing EMT can evade from their original epithelial layer. Recently, an increasing number of studies have described ROS as the main cause of EMT in cancer cells, finally promoting cancer cell invasion and metastasis (Fig. 3) 66.

Oxidative stress is an essential factor causing cell-cell junction dissociation 67. The elevated levels of ROS can activate nuclear factor-κB (NF-κB) and promote the expression of transcription factor Snail, which downregulates the expression of epithelial cadherin (E-cadherin) and promotes the expression of neural cadherin and vimentin 68, 69, thus resulting in the disruption of cell-cell junctions and triggering the EMT process 23. Moreover, ROS can promote the expression of transforming growth factor-β (TGF-β), and TGF-β favors the formation of trimeric drosophila mothers against decapentaplegic protein (SMAD) complexes 23, 70. The SMAD complexes can translocate to the nucleus and activate mesenchymal genes as well as* Snail*, *Twist*, *Slug*, and *ZEB1* genes, in turn enhancing the expression of vimentin and fibronectin and repressing E-cadherin expression 70. Besides, the upregulation of ROS also promotes HIF-1α-induced transcription of Snail, thus leading to the repression of E-cadherin 71. Furthermore, ROS also stabilize nuclear factor erythroid 2-related factor 2 (NRF2) via the degradation of Kelch-like ECH-associated protein 1 (KEAP1) to enhance the transactivation of Notch homolog 1, translocation-associated (Drosophila) (NOTHC1), promoting the NOTHC1 intracellular domain translocation to the nucleus and leading to Snail transcription, thereby promoting the EMT of cancer cells and favoring cancer cell invasion and metastasis 72. In addition to the aforementioned signaling, the ROS-activated PI3K/Akt signaling pathway can inhibit glycogen synthase kinase 3β (GSK-3β), the activation of which is involved in the proteasomal degradation of Snail 23, 73, 74. The inhibition of GSK-3β can promote the nuclear-translocation of β-catenin, following which nuclear β-catenin binds with TCF/LEF and enhances the transcription of vimentin and slug 75, finally inducing EMT. ROS can lead to the loss of cell-cell junctions via NF-κB, TGF-β, HIF-1α, NRF2, and PI3K/Akt signaling, inducing transition to the mesenchymal phenotype.

ROS also cause cytoskeleton remodeling, favoring cancer cell migration, and thus promoting the EMT process. The cytoskeleton consists of actin filaments, intermediate filaments, and microtubules, and it is important to maintain the cell shape 76. Actin filaments are twisted by two strands of actin polymer fiber actin (F-actin), and F-actin is polymerized by actin monomer spherical actin (G-actin). The actin filament is a dynamic structure responsible for cell migration capacity. ROS can regulate polymerization and depolymerization between G-actin and F-actin to achieve dynamic actin reorganization. ROS can oxidize 14-3-3ζ, which inactivates slingshot-1L (SSH-1L), thus alleviating the inhibition of SSH-1L 77. SSH-1L ultimately results in the dephosphorylation and activation of cellular cofilin; the activation of which leads to the depolymerization of F-actin, thus providing actin monomers to further polymerize 77, 78. Besides, ROS induce Arp2/3 recruitment, which can nucleate actin monomers to assemble a new actin filament meshwork 78, 79. ROS-regulated cofilin and Arp2/3 maintain actin treadmilling and drive cells to form actin-rich membrane projections called lamellipodia, which promote cytoskeletal extension 78, 79. Subsequently, ROS increase the formation of actin stress fibers 80, which are microfilament bundles formed by actin filaments 81. Additionally, ROS also inhibit protein tyrosine phosphatase to sustain the phosphorylation of focal adhesion kinase (FAK) 23. On the one hand, FAK can lead to focal adhesion, and actin stress fibers located on the inside of the cell membrane adhere to the focal adhesion spot 82. On the other hand, FAK can recruit talin, an actin-binding protein, which reacts with the extracellular domains of integrins 83. Integrins are cell surface adhesion molecules, and integrin α7β1 modified by ROS is a link to laminin-111 in the ECM 84. Thus, a connection exists between the ECM and intracellular actin cytoskeleton, which promotes cell migration.

During the EMT process, cancer cells acquire an invasive phenotype by recognizing and attaching to the ECM to degrade the basement membrane and ECM proper. Several studies have indicated that ROS can cause ECM degradation by regulating proteolytic enzymes, such as matrix metalloproteinases (MMPs) and serine proteases, both of which are thought to be involved in tumor malignancy 85, 86. ROS mainly promote activator protein-1 (AP-1) and NF-κB-induced transcription of MMPs, causing protein matrix degradation 87, 88. Besides, ROS activate mitogen-activated protein kinase (MAPK), AP-1, and NF-κB signaling pathways to promote the expression of urokinase plasminogen activator (uPA) and uPA receptor (uPAR) 85, 86, 89, 90. ROS also enhance the binding of mRNA-stabilizing factor Hu antigen R (HuR) with AU-rich element (ARE)^uPA^ to promote the expression of uPA and uPAR 91. Activated uPA, a serine protease, leads to the transformation of plasminogen into plasmin, resulting in MMP activation, ECM degradation, and eventual cancer cell invasion 23, 86.

## Potential mechanisms underlying the anti-cancer effects of ROS

### Anti-cancer effects of ROS

**Fig 4:**
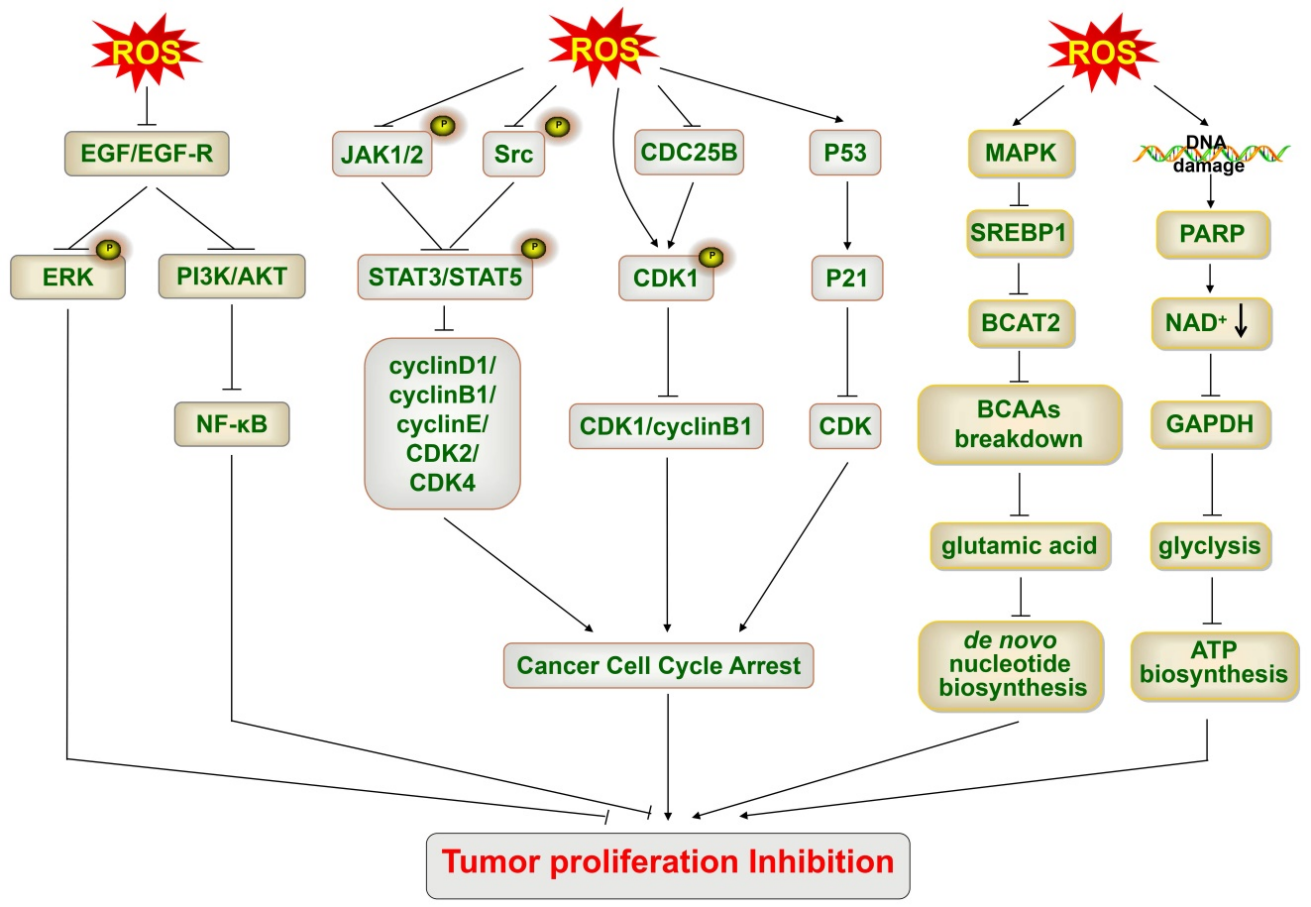
** The accumulation of ROS leads to cancer cell growth inhibition.** The accumulation of ROS inhibits EGF/EGF-R, then blocks ERK and PI3K/Akt/NF-κB signaling, and inhibits cancer cell proliferation. The accumulation of ROS decreases the phosphorylation of JAK1/2 and Src, thus blocking the phosphorylation of STAT3/STAT5 and downregulating the expression of cyclin D1, cyclin B1, cyclin E, CDK2 and CDK4, and finally leading to cell cycle arrest. ROS phosphorylate CDK1 (inactive) directly or indirectly by inactivating cdc25B, thus suppressing the CDK1/cyclin B complex and resulting in cell cycle arrest. ROS activate p53 and then trigger p21, an inhibitor of most CDKs, thus finally leading to cell cycle arrest. ROS accumulation activates MAPK, suppresses SREBP1 and inhibits BCAT2, thus decreasing the breakdown of BCAAs and the synthesis of glutamic acid, and finally leading to a decrease in de novo nucleotide biosynthesis. ROS accumulation induces DNA damage and activates PARP, thereby leading to a decrease in NAD^+^, causing the inactivation of GAPDH and inhibition of glycolysis, and ultimately leading to the depletion of ATP and inducing an energetic crisis. EGF-R, EGF receptor; SREBP1, sterol regulatory element-binding protein 1; BCAT2, branched-chain amino acid transaminase 2; BCAAs, branched-chain amino acids; GAPDH, glyceraldehyde 3-phosphate dehydrogenase; NAD+, nicotinamide adenine dinucleotide oxidized form.

**Fig 5:**
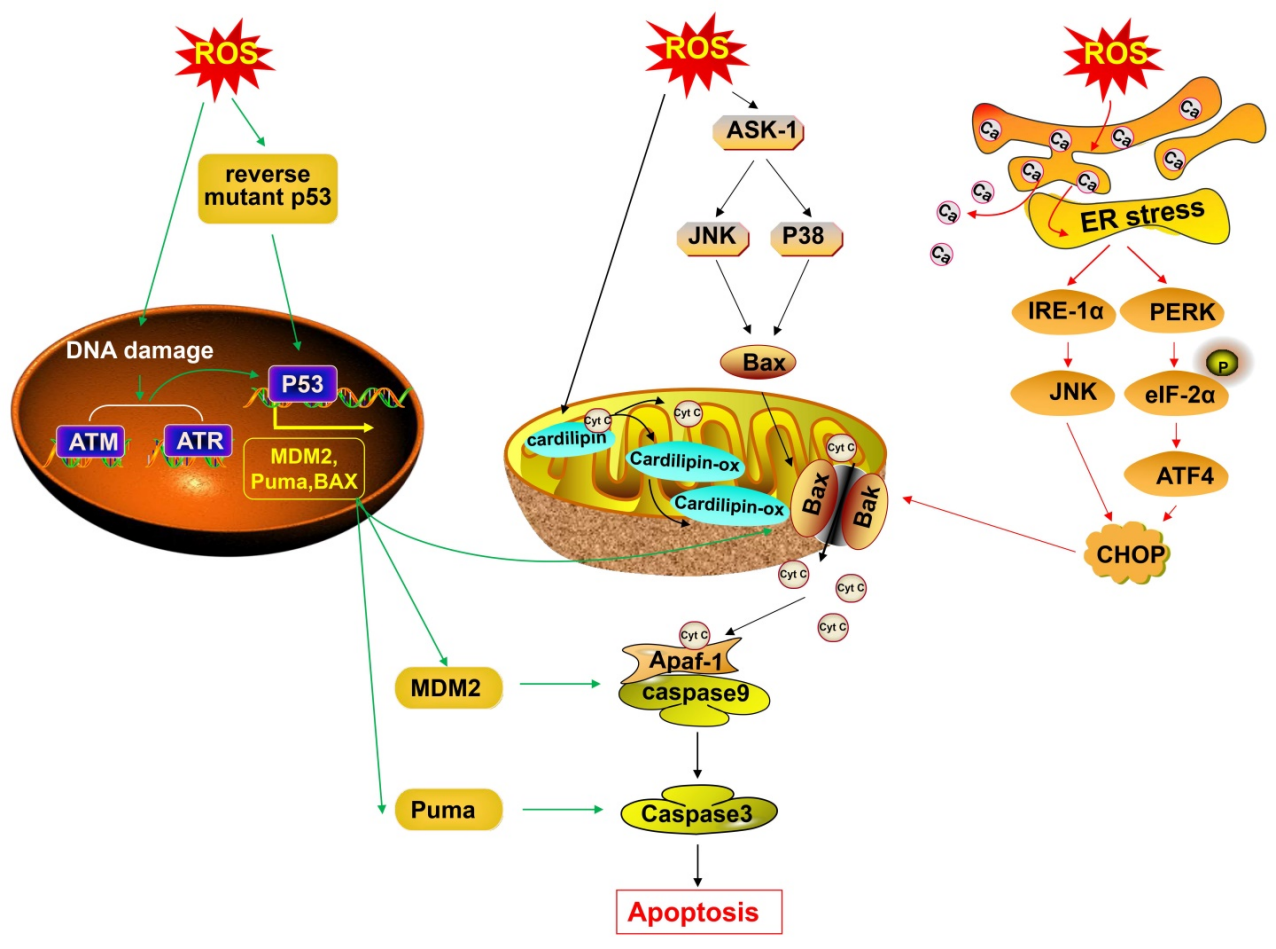
** The accumulation of ROS causes cancer cell apoptosis.** Accumulated-ROS induce DNA damage, thus causing the activation of ATM and ATR, and activating P53. Abundant ROS reactivate mutant P53 protein, and P53 subsequently promotes the expression of pro-apoptotic proteins, BAX, MDM2 and Puma, thus subsequently leading to cancer cell apoptosis. ROS oxidize cardiolipin, thereby resulting in Cyt c detachment from the membrane. Oxidized cardiolipin translocates to the outer membrane and recruits the pro-apoptotic protein BAX to the mitochondrial membrane, thus enhancing MOMP and generating Cyt c-traversable pores. ROS also activate ASK/JNK and ASK/P38 axis and promote the release of Cyt c in a manner dependent on BAX. Cyt c interacts with Apaf-1, thus resulting in apoptosome formation, leading to caspase-9 and caspase-3/7 activation, and causing cancer cell apoptosis. The accumulation ROS also causes cancer cell apoptosis via the ER stress pathway through the PERK/eIF2α/ATF-4/CHOP axis and IRE1α/JNK/CHOP axis. MDM 2, murine double minute 2; Puma, p53 upregulated modulator of apoptosis; Apaf1, apoptotic protease activating factor 1; PERK, protein kinase-like ER kinase; IRE1α, inositol-requiring enzyme-1α; ATF4, activating transcription factor-4.

In the last few years, several studies have demonstrated a dual role of ROS (“pro-tumor” and “anti-cancer”) 92. In the early stage, high levels of ROS are crucial in promoting tumor initiation and tumor progression, as discussed earlier. However, the massive accumulation of ROS plays an antioncogenic role in cancer. Extensive accumulation of ROS can inhibit tumor cell proliferation 93 and lead to cancer cell death, which can be explained by the activation of ER stress-, mitochondrial-, P53- apoptotic pathways and the ferroptosis pathway in cancer 94-96 (Fig. 4, Fig. 5). The anti-cancer effects of ROS can provide an additional avenue for cancer treatment. Thus, it is of great interest to discuss the potential mechanisms of the anti-cancer effects of ROS.

### Potential mechanisms of anti-cancer effects of ROS

#### ROS inhibit cancer cell proliferation

##### ROS inhibit the proliferation signaling pathway

Cancer cells are characteristic of uncontrolled cell proliferation and cell growth. ROS can inhibit cancer cell growth by inhibiting cell proliferation. Epidermal growth factor (EGF) and EGF receptor (EGFR) signaling play critical roles in tumor growth. When ROS levels exceed the self-regulating ability of cancer cells, ROS directly decrease the expression of EGF and EGFR and markedly inhibit the phosphorylation of EGFR; as a result, downstream cell proliferation signaling molecules are inhibited, such as ERK and PI3K/Akt 97-99. Vitamin C has been reported to promote the production of ROS and inhibit the phosphorylation of ERK by decreasing EGF release and EGF receptor phosphorylation, thus suppressing the proliferation of thyroid cancer cells 99. Similarly, Koumine also promotes the production of ROS, which inhibits the phosphorylation of ERK, consequently suppressing the proliferation of hepatocellular carcinoma cells 93. In line with this, the administration of H_2_O_2_ can impair the phosphorylation of ERK1/2 in breast cancer cells in a dose-dependent manner, thus attenuating cancer cell proliferation 100. Additionally, excessive ROS inhibit EGF-induced EGFR mediated PI3K/Akt signaling and ultimately block androgen-independent prostate cancer cell proliferation 97. In addition, ROS block the PI3K/Akt/NF-κB signaling pathway, which subsequently leads to the inhibition of the proliferation of human nonsmall cell lung cancer, A549 cells; the same mechanism associated with oenothein B-mediated suppression of cancer cell growth 98.

##### ROS inhibit the cell cycle

Somatic cell proliferation mainly depends on mitosis. Somatic cell mitosis is periodic and referred to as the cell cycle. The cell cycle is composed of four phases: the gap 1 (G1) phase, synthesis (S) phase, G2 phase, and mitosis (M) phase 101. A specific cyclin can bind to a specific cyclin-dependent kinase (CDK) to form a complex, regulating different phases of the cell cycle. CDK4 and CDK6 can bind to cyclin D to regulate the early G1 phase 102. CDK2 binds to cyclin E to cause S-phase initiation, and later CDK2/cyclin A complex formation promotes DNA replication in the S phase 101. CDK1/cyclin B complex can regulate G2 to M phase transition 101. The accumulation of ROS in multiple myeloma cells reduces the phosphorylation of Janus kinase (JAK)1, JAK2, and Src, thus blocking the phosphorylation of signal transducers and activators of transcription3 (STAT)/STAT5 and downregulating the expression of cyclin D1, cyclin B1, cyclin E, CDK2, and CDK4, finally leading to cell cycle arrest and attenuating tumor growth 103. The overexpression of ROS in human nonsmall cell lung cancer cells also promotes CDK1 phosphorylation and suppression of cyclin B1 and CDK1 expression, subsequently blocking CDK1/cyclin B1 complex formation and leading to G2/M phase arrest 104. The accumulation of ROS also controls cell cycle regulatory molecules, such as cell division cycle (cdc)25 and CDK inhibitors, to suppress tumor growth 101. LGH00031 accelerates cellular ROS production to inactivate cdc25B, which dephosphorylates tyrosine15 of CDK1 to activate the CDK1/cyclin B complex, resulting in cell cycle arrest and inhibition of cancer cell growth 105. Physalin A increases the levels of intracellular ROS to activate p53 and then trigger p21, an inhibitor of most CDKs, finally leading to cell cycle arrest of human nonsmall cell lung cancer cells 104.

##### ROS suppress the biosynthesis of nucleotides and ATP

Sufficient nucleotides and ATP are required to meet the needs of highly mitotic cells. The accumulation of ROS can impair the ability of cancer cells to synthesize nucleotides and ATP, thus failing to meet their demand for cell proliferation 106, 107. ROS accumulation mediates the activation of the MAPK pathway in pancreatic cancer cells, suppresses sterol regulatory element-binding protein 1, and inhibits its target gene branched-chain amino acid transaminase 2, causing a decrease in the breakdown of branched-chain amino acids and thus reducing the synthesis of glutamic acid, finally leading to a decrease in *de novo* nucleotide biosynthesis 106. Moreover, ROS accumulation inhibits glycolysis by the inactivation of glyceraldehyde 3-phosphate dehydrogenase via both direct post-translational modifications and depletion of the nicotinamide adenine dinucleotide oxidized form, which is consumed by PARP activated by ROS-induced DNA damage, ultimately leading to the depletion of ATP, inducing an energetic crisis, and finally killing *KRAS* and *BRAF* mutant colorectal cancer cells 107.

#### ROS induce tumor cell apoptosis

##### ER stress-mediated apoptosis

The accumulation of ROS in cancer cells can cause apoptosis via the ER stress-mediated pathway. ROS can cause the release of Ca^2+^ ions from the ER lumen, subsequently resulting in defects in ER chaperones and other proteins, thus leading to ER stress with the accumulation of unfolded/misfolded proteins 108. ER stress caused by the accumulation of ROS induces the phosphorylation of eIF2α via RNA-dependent protein kinase-like ER kinase, allowing the preferential translation of activating transcription factor-4, which in turn mediates the induction of pro-apoptotic C/EBP homologous protein (CHOP), leading to cancer cell apoptosis 94, 109. Moreover, ROS-induced ER stress can mediate cancer cell apoptosis via the inositol-requiring enzyme-1α/ c-Jun NH(2)-terminal kinase (JNK)/CHOP pathway 110. Persistent ER stress initiates apoptotic cascades that play fundamental roles in the mechanism underlying the anticancer properties of ROS 109, 111-113. The alantolactone-mediated accumulation of ROS breaks the imbalance of intracellular redox status in triple-negative breast cancer cell lines, increasing the levels of unfolded proteins in the ER and resulting in ER stress, subsequently leading to cancer cell apoptosis 113. Additionally, β-phenethyl isothiocyanate and curcumin also trigger ER stress-mediated apoptosis via ROS accumulation to treat cancers 111, 112.

##### Mitochondrial apoptotic pathway

In addition to ER stress-mediated apoptosis, the mitochondrial apoptotic pathway regulated by ROS accumulation has anticancer effects. The mechanisms reported so far suggest that the accumulation of ROS can drive the release of pro-apoptotic factor cytochrome c (Cyt c) from the mitochondria to the cytosol, which is a key step in the mitochondrial pathway, finally inducing cancer cell apoptosis 114. Cyt c is anchored to the outer surface of the mitochondrial inner membrane via combination with cardiolipin, a mitochondria-specific phospholipid 115. The release of Cyt c relies on disrupting the interaction of Cyt c with cardiolipin and enhancing mitochondrial outer membrane permeabilization (MOMP), which is regulated by pro-apoptotic proteins Bcl-2-associated X protein (BAX) and Bcl-2-antagonist killer (BAK) 116. ROS can oxidize cardiolipin, which results in Cyt c detachment from the membrane 116. The oxidized cardiolipin translocates to the outer membrane and helps to recruit pro-apoptotic protein BAX to the mitochondrial membrane, thus enhancing MOMP and generating Cyt c-traversable pores 117. Besides, ROS can activate apoptosis signal-regulating kinase 1, a member of the mitogen-activated protein kinase kinase kinase superfamily, to activate JNK and P38 MAPK, the activation of which promotes the release of Cyt c on the mitochondrial inner membrane in a manner dependent on BAX 118-120. Moreover, the accumulation of ROS can mediate the ER stress pathway and then activate the expression of pro-apoptotic protein Noxa 136, 137. Activated Noxa translocates into the mitochondria and subsequently inhibits anti-apoptotic proteins, thereby reversing the inhibition of BAX and BAK, forming MOMP, and finally promoting the release of Cyt c 121, 122. Then, Cyt c interacts with apoptotic protease activating factor 1 to form an apoptosome, leading to caspase-9 and caspase-3 activation and subsequent cell apoptosis 123. The administration of α-hederin can induce the overexpression of ROS and activate the mitochondrial apoptotic pathway, finally triggering the apoptosis of gastric cancer cells 95. Similarly, *in vitro* studies have found that metal thiourea complexes can work as a new antitumor metallodrug to kill HeLa cells via a burst of the ROS-regulated mitochondria apoptotic pathway 114.

##### The P53 apoptotic pathway

Most cancers have abrogation of P53 function with a wide range of mutation frequencies 124. The hot mutations of P53 are DNA contact mutation (P53R273H), which impairs P53 contact with DNA, and conformational mutation (P53R175H), which affects the stability of the DNA-binding domain, resulting in the loss of function 124. Notably, ROS can act as an intermediate medium to reverse the abrogated P53 function. Piperlongumine reverses the R273H mutant P53 protein by overproducing ROS and leads to a restoration of the functional status of mutant P53 in human colon cancer cells, inducing the transcription of the P53 target gene, murine double minute 2, and BAX and finally resulting in cancer cell apoptosis 125. R-goniothalamin-induced abundant ROS reactivate the R175H mutant P53 protein in human breast cancer cells, and then P53 promotes the expression of pro-apoptotic proteins: p21cip1, BAX, and p53 upregulated modulator of apoptosis, causing tumor cell apoptosis 96. ROS not only enhance the transcription effect of P53 but also translocate P53 to mitochondria, contributing to BAX mitochondrial recruitment and mitochondrial Cyt c release, thus leading to cancer cell apoptosis 126. In addition, the accumulation of ROS can trigger cancer cell DNA damage, and ROS-induced DNA damage causes the activation of DNA damage sensors and regulators such as ataxia telangiectasia mutated (ATM) and ataxia telangiectasia and Rad3-related (ATR), thus activating P53 and subsequently leading to cancer cell apoptosis 127, 128.

#### ROS-induced ferroptosis

Ferroptosis is a form of iron-dependent cell death characterized by the lethal accumulation of lipid-based ROS and excessive accumulation of lipid peroxidation 129*.* Excessive iron from aberrant iron metabolism and the loss of cellular antioxidant capacity can cause the accumulation of ROS, thereby inducing catastrophic alterations in cellular redox metabolism and leading to massive lipid peroxidation in the plasma membrane and loss of cell viability, and eventually ferroptosis 130, 131. Cancer cells are more vulnerable to ferroptosis than noncancerous cells, owing to the powerful iron dependency feature 131*.* ROS-induced ferroptosis is one of the most important means of inhibiting cancer. Several ferroptosis inducing drugs have been used to treat cancer. Erastin, artesunate, and sorafenib have been reported to inactivate glutathione peroxidase 4, an important antioxidant, via inhibiting the synthesis of glutathione (GSH), thereby inducing oxidative stress in cancer cells and ultimately leading to ferroptosis 129, 132, 133. Ferroptosis inducers have led to high expectations regarding the potential of ferroptosis to be a new promising method of killing cancer cells.

## Self-adaption system in cancer

**Fig 6:**
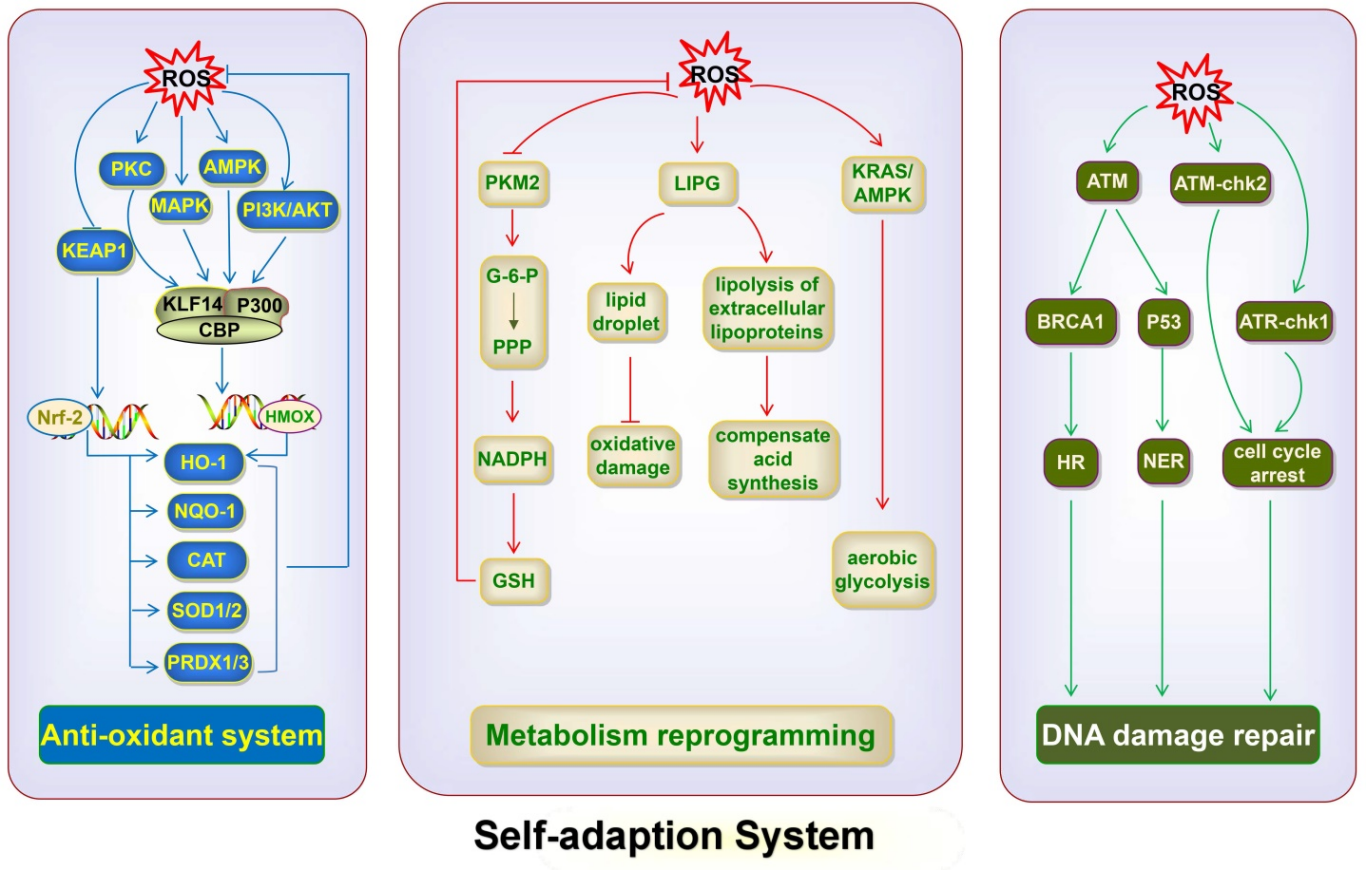
** ROS utilize the antioxidant system, DNA damage repair pathway, and metabolic reprogramming to inhibit the accumulation of ROS and repair ROS-induced damage.** ROS activate NRF2, thereby promoting the expression of NQO1, CAT, SOD1/2 and PRDX1/3, and decreasing ROS levels. ROS activate the expression of KLF14 via the PI3K/Akt, p42/p44 MAPK, AMPK, and PKC pathways; KLF14 then couples with p300 and CBP, and promotes the expression of HO-1, which restores cellular redox balance. ROS accumulation inhibits PKM2, thus leading to G-6-P flux into the PPP and generating sufficient NADPH to produce GSH, thereby maintaining ROS homeostasis. ROS trigger LIPG upregulation and induce the lipolysis of extracellular lipoproteins to compensate for the ROS-impeded fatty acid synthesis pathway. The upregulated LIPG also protects against oxidative damage via the accumulation of intracellular lipid droplets. ROS activate KRAS/AMPK signaling, thus inducing metabolic reprogramming toward aerobic glycolysis. ROS activate ATM, which in turn phosphorylates BRCA1 and triggers HR for DNA repair and activation of P53, which in turn triggers the NER pathway. ROS activate the ATM-chk2 and ATR-chk1 axis, thereby causing cell cycle arrest of cancer cells, preventing the cells from undergoing mitosis and favoring DNA repair. NQO1, NADH dehydrogenase, quinone 1; CAT, catalase; PKC, protein kinase C; G-6-P, glucose-6-phosphate; PPP, pentose phosphate pathway.

As mentioned earlier, the massive accumulation of ROS is generally detrimental to cancer cells. ROS can inhibit cancer cell growth by suppressing the synthesis of ATP and nucleotides, leading to cell cycle arrest and blocking cancer cell proliferation. ROS also cause cancer cell death through several apoptotic pathways. However, cancer cells use the self-adaption system, including the antioxidant system, DNA damage repair pathway, and metabolism reprogramming, to inhibit the accumulation of ROS and ameliorate ROS-induced damage, so as to maintain their development and survival (Fig. 6).

### Antioxidant system

The antioxidant system is essential to maintain cellular redox balance. Accumulating evidence shows that NRF2 is pivotal in antioxidant responses. Importantly, it is a key gene for cancer cells to maintain oxidative balance. The target antioxidant genes of NRF2 include reduced nicotinamide adenine dinucleotide dehydrogenase, quinone 1, SOD1/2, catalase, heme oxygenase-1 (HO-1), thioredoxin reductase 1, peroxiredoxin (PRDX)1, and PRDX3, all of which are antioxidant enzymes that reduce ROS levels 134. Under oxidative stress, ROS can cause the binding of 5′-adenosine monophosphate-activated protein kinase (AMPK) to HCC-specific fructokinase A (KHK-A), induce KHK-A phosphorylation of serine at position 80, and subsequently phosphorylate p62 at S28 135. Consequently, p62 oligomerization occurs and the aggregation of p62 with KEAP1 that inhibits NRF2 is enhanced, thereby increasing nuclear translocation and activation of NRF2 to alleviate ROS, and finally promoting the development of HCC 135. Besides, accumulated ROS lead to lactate dehydrogenase A nuclear translocation to promote the production of α-hydroxybutyrate (α-HB) in HPV-induced cervical tumors 9. Then, α-HB triggers the disruptor of telomeric silencing 1-like-mediated histone H3K79 hypermethylation, inducing the activation of NRF2 to control cellular redox balance 9. Cancer cells also use other factors, besides NRF2, to control antioxidant activity. Oxidative stress activates the expression of Krüppel-like factor 14 (KLF14) via multiple pathways, including the PI3K/Akt, p42/p44 MAPK, AMPK, and protein kinase C pathways, in castration-resistant prostate cancer. Then, KLF14 couples with p300 and CREB-binding protein (CBP) to enhance the transcriptional activation of heme oxygenase 1, which encodes the antioxidant enzyme HO-1 that helps cancer cells restore cellular redox balance under androgen-depleted conditions, resulting in cancer cell survival 136.

### DNA damage repair pathway

Cancer cells use the DNA damage repair pathway to adapt to DNA damage caused by chemotherapy and radiotherapy (RT), finally leading to therapeutic resistance. The key regulators of the DNA damage repair process are ATM and ATR 137. ATM and ATR can recognize DNA lesions and phosphorylate downstream molecules to transmit DNA damage signals, leading to cell cycle arrest and finally completing DNA repair 138. Under oxidative stress, ROS regulate the activation of ATM, which recognizes DNA damage 138. ATM triggers signal transduction of checkpoint kinase 2 (Chk2), and the ATM-Chk2 axis causes cell cycle arrest of cancer cells, preventing the cells from undergoing mitosis and favoring DNA repair 139. Besides, ATM can both phosphorylate BRCA1, which is required for HR 140, and activate P53, which can trigger the NER pathway 141, 142, finally leading to DNA repair. Additionally, ROS can induce the phosphorylation of ATR, which in turns phosphorylate Chk1, resulting in the cell cycle arrest of cancer cells and better repair of DNA damage 143, 144. The overproduction of ROS activates the ATR-Chk1 axis, resulting in resistance to cisplatin in ovarian cancer cells, and the inhibition of ATR and Chk1 reverses chemotherapeutic resistance 145. The findings provided strong evidence that accumulated ROS triggered the DNA damage repair pathway to resist the ROS-caused damage in cancer cells.

### Metabolism reprogramming

Metabolism reprogramming is also a critical way to resist ROS-induced inhibition of cancer cell development, besides antioxidant system and DNA damage repair pathway. Cancer cells can change their metabolic pathway to maintain the oxidative balance and their nutrient needs and cause therapeutic resistance. Accumulated ROS can trigger metabolism reprogramming to reduce the levels of ROS in cancer cells. The accumulation of ROS inhibits glycolytic enzyme pyruvate kinase M2 (PKM2) via the oxidation of Cys358 in human lung cancer cells 146. The inhibition of PKM2 leads to glucose-6-phosphate flux into the pentose phosphate pathway, which generates sufficient nicotinamide adenine dinucleotide phosphate oxidase to produce GSH, thus maintaining ROS homeostasis and favoring cancer cell survival 146. The change in the abundance of selected lipids can promote tumor growth and progression by eliminating ROS in colorectal cancer development 147. The accumulation of ROS can also trigger metabolism reprogramming to maintain the nutritional needs of cancer cells. A study has shown that severe oxidative stress triggers endothelial lipase (LIPG) upregulation, which induces the lipolysis of extracellular lipoproteins, to compensate the ROS-impeded fatty acid synthesis pathway in breast cancer cells, thus supporting tumor progression 148. The upregulated LIPG also results in the accumulation of intracellular lipid droplets in breast cancer cells, which protects against oxidative damage, finally supporting survival 148. Additionally, metabolic reprogramming leads to tumor treatment tolerance. Gemcitabine-induced ROS activate KRAS/AMPK signaling, which induces metabolic reprogramming toward aerobic glycolysis, enhancing stem-like cell properties in pancreatic cancer 149. Oxidative stress-induced metabolic reprogramming is one of the most important reasons for the therapeutic resistance of pancreatic cancer because cancer stem cells are insensitive to external physical and chemical factors that kill tumor cells 149.

## Scientific significance of the dual role of ROS in cancer

The incidence of cancer has increased gradually, and cancer acts as the leading cause of death among humans. Fortunately, a large number of therapeutics have been developed. Increased levels of ROS can suppress tumor cell growth and lead to apoptosis (Table 1). Recent studies show that a large number of therapeutic regimens to treat cancer depend on the elevation of ROS levels. However, ROS have a dual role in cancer, and cancer cells own a self-adaption system to resist the harmful effects of ROS. In normal cells or during early stages of cancer, the upregulation of ROS can cause gene mutations and tumor initiation that favor tumor invasion and metastasis (Table 2). If the ROS levels increase significantly, the therapeutic effect cannot be achieved; instead, ROS promote the development of cancer. Thus, different methods should be used to treat cancers in different stages. In the early carcinogenesis stage, attention should be paid to the therapeutic regimens depending on the downregulation of ROS. Nanoparticles (NPs) containing ROS-scavenging nitroxide radicals are synthesized to abrogate ROS-mediated cell proliferation and metastasis to attenuate cancers 150. However, in advanced cancer stages, enhancing the production of ROS to kill tumor cells and inhibiting the antioxidant system, DNA damage repair pathway, and metabolism reprogramming should be considered, as inhibition can effectively resolve therapeutic resistance. During the course of cancer treatment, considering the different roles of ROS in different stages of cancer can effectively increase the preventive and anticancer effects.

## Clinical application of the dual role of ROS in cancer

Many traditional chemotherapeutic agents used in clinical settings, such as doxorubicin (DOX) and pirarubicin, can induce intracellular ROS accumulation and trigger the mitochondrial apoptotic pathway, finally leading to cancer cell death 151. However, some chemotherapeutic agents, such as anthracyclines, have cytotoxic effects on normal cells 152. Many laboratories are committed to finding natural drug ingredients with lower cytotoxic effects on normal cells to treat malignant tumors 153. Galangin (GG), a naturally active flavonoid extracted primarily from the root of *Alpinia officinarum* Hance, can kill cancer cells without causing cytotoxicity in normal cells 154. Experiments have demonstrated that the natural drug GG, combined with tumor necrosis factor-related apoptosis-inducing ligand, enhances ROS production, leading to the apoptosis of human breast cancer cells 155. Other natural drugs, such as curcumin and parthenolide, also increase the levels of ROS to cause cancer cell apoptosis 112, 156. Besides chemotherapy, RT also becomes an important means to treat malignant tumors in clinic. RT uses ionizing radiation to promote the production of ROS to treat cancer 157. Ionizing radiation not only leads to the radiolysis of water to produce large amounts of ROS 158 but also promotes the expression of NOX1 to overexpress ROS 159. The ROS produced react with DNA, finally leading to DNA strand breaks 160. Failure to perform normal functions after DNA strand breaks causes cancer cell death 161.

However, the emergence of treatment resistance hinders the efficacy of cancer treatment. The antioxidant system of cancer cells is important in tumor treatment resistance. High mucin 1 C-terminal subunit expression in urothelial carcinoma cells stabilizes the expression of x-cystine/glutamate transporter and increases intracellular GSH levels, thus causing cisplatin resistance by urothelial carcinoma 162. Also, after radiation treatment, free radical species can stabilize HIF1, the master regulator of oxygen homeostasis, finally buffering ROS generated by radiation, and hence mitigating the effect of RT 163. It is important to find drugs that inhibit the function of the antioxidant system to solve the problem. Fortunately, a study has shown that piperlongumine can inhibit glutathione and thioredoxin systems, thus reducing ROS clearance and enhancing the radioresponse of colorectal cancer cells 164. Besides, the activation of the DNA damage repair pathway can counteract DNA damage caused by chemotherapy and RT, which is one of the main causes of treatment resistance. Radiation can upregulate the expression of the *ATM* gene, which triggers DNA damage repair, finally mitigating the ROS-induced DNA lesions 165, 166. Obviously, inhibiting the RT-induced DNA damage repair pathway is a useful way to solve the problems. Another study has reported that the combination treatment of resveratrol and capsaicin, besides RT, can inhibit ATM activation and thus radiosensitize pancreatic tumor cells toward cell death 167.

New therapies have partly resolved the treatment resistance to RT and chemotherapy. However, the biggest problem at present is the inability to deliver targeted therapies. Radiotherapy and chemotherapy can induce ROS production in nontargeted tissues, and the high concentrations of ROS can damage normal cells and even cause carcinogenesis. Therefore, targeted treatments have become a hot research spot, and new therapies have emerged based on the requirement. The advent of photodynamic therapy (PDT) has enabled the precise and effective treatment of tumors. PDT is an outstandingly selective, effective, and noninvasive cancer therapeutic methodology comprising a light source, photosensitizer, and oxygen 168. A specific wavelength of laser light excites the photosensitizer absorbed by the tissue, and the excited-state photosensitizer transfers energy to the surrounding oxygen molecules, generating a highly active singlet oxygen (an ROS), which can kill cancer cells precisely. However, hypoxia often occurs in solid tumors with very low oxygen concentrations 169. Under hypoxic conditions, the generation of singlet oxygen is reduced because of the low oxygen level, and hence the therapeutic efficacy of PDT in clinical treatment is greatly suppressed 170. Initiator-loaded gold nanocages, as the free radical generator, can generate ROS under different oxygen tensions on exposure to near-infrared light irradiation 171, effectively solving the problem of PDT resistance caused by hypoxia.

The application of nanoparticles has opened new avenues to develop cancer therapies, with the advancement in nanotechnological techniques and further investigations on nanoparticles in biomedical sciences. NPs can be divided into inorganic nanomaterials such as iron oxide NPs (IO NPs) and cerium oxide NPs (CONPs), and organic nanomaterials such as polymeric micelles 172-174. It is possible to further reduce the side effects and improve the efficacy of tumor treatment due to the selective targeting capabilities and superior efficacy of NPs 175. IO NPs, serving as a drug vehicle, conjugate with anticancer drug DOX, which can promote the production of ROS, forming magneto-sensitive NPs 173. These NPs can selectively target using magnetic fields in cancer treatment 173. IO NPs can also link with linoleic acid hydroperoxide (LAHP). Under the acidic-pH condition, Fe^2+^ ions are released from IO NPs and react with LAHP, triggering the generation of singlet oxygen (^1^O_2_), which can kill cancer cells 176. IO-LAHP NPs can produce tumor-specific ^1^O_2_ to treat cancer due to the overall acidic environment of solid tumors 177, which contribute to targeted treatment 176. Besides selectively killing cancer cells, NPs can combine with other therapies, such as RT and PDT, to enhance the efficacy of cancer treatment. Experiments have demonstrated that CONP pretreatment facilitates the production of RT-induced ROS in human pancreatic cancer cells 174, indicating that CONPs can serve as an RT sensitizer to improve pancreatic cancer treatment. Likewise, polymeric micelles are used in PDT for cancer therapy. Polymeric micelles, hemoglobin (Hb), and zinc phthalocyanine form an Hb-conjugated photosensitizer carrier, which has an oxygen self-compensating ability in PDT 172. Under light irradiation, the Hb-conjugated photosensitizer carrier can induce the production of more ROS, thus causing cancer cell death 172. NPs can target and control the release of ROS to reduce side effects. They can also combine with other therapies to improve the efficacy of cancer treatment. Hence, NPs have broad application prospects and development space.

## Summary and perspectives

Anticancer treatment and early cancer prevention have become hot topics in medical research due to the increased morbidity and mortality of cancer. Targeting ROS has become an efficient way to treat cancer. Gaining a deeper understanding of the various mechanisms of ROS can provide better insights into cancer treatment. This review illustrated the dual role of ROS in promoting cancer occurrence and development and inhibiting cancer growth. In the early stages, elevated levels of ROS act as pro-tumorigenesis factors, which can induce DNA mutation and EMT, thus favoring cancer initiation, development, invasion, and metastasis. Besides, a high level of ROS inhibits T cells and NK cells and promotes the recruitment and M2 polarization of macrophages; consequently, cancer cells escape immune surveillance and immune defense. Interestingly, excessively accumulated-ROS repress cancer cell growth by inhibiting the production of essential substances needed for cell life activities, such as nucleotides and ATP, causing cell cycle arrest and blocking cancer cell proliferation. ROS also induce cancer cell death by activating ER stress-, mitochondrial-, and P53-apoptotic pathways and the ferroptosis pathway. Of note, tumor cells can establish a self-adaption system (antioxidant response, DNA damage repair pathway, and metabolism reprogramming) to deal with massively accumulated ROS and the damage they cause. Furthermore, the review discussed the methods and development of cancer treatment in recent years and also focused on therapeutics using ROS to treat cancer. The characterization of the dual role of ROS in cancer and the self-adaptive ability of cancer cells may provide a new idea for the prevention of carcinogenesis and the development of new therapeutic regimens for cancers.

An increasing number of studies have shown that various cancer cells always have high levels of ROS 4. ROS can promote the development of cancer by inducing gene mutation, mitochondrial dysfunction, and EMT, and activating anti-apoptotic pathways. In particular, ROS can inhibit the function of immune cells. Eventually, cancer cells escape from immune surveillance and immune defense. Interestingly, age-related thymic involution is closely connected with elevated levels of ROS 178. A previous study showed that a murine oxidative stress model via ozone inhalation exhibited accelerated thymic involution 179. Consistent with this finding, the administration of antioxidant compounds can restore the size of the thymus 180. The thymus is a site for T-cell differentiation and development. The thymic involution leads to the decline of T-cell output with decreased immune function 181. A mathematical model of cancer incidence has shown that the aging immune system leads to an increased risk of cancer 182. Therefore, it is speculated that ROS-mediated thymic involution is closely associated with carcinogenesis, thus providing a new basis for immune remodeling to treat cancer. However, the specific mechanism of ROS causing thymus involution to regulate cancer development is not clear.

## Figures and Tables

**Table 1:** The anti-cancer effects of ROS

Function	Result	Effect
Decrease EGF and EGFR expression	Inhibit the proliferation signaling pathway	Inhibit cancer cell proliferation
Inhibit EGFR phosphorylation		
Reduce JAK1, JAK2, and Src phosphorylation	Cell cycle arrest	
Promote CDK1 phosphorylation		
Suppress cyclin B1 and CDK1 expression		
Inactivate cdc25B,		
Activate p53		
Activate MAPK	Decrease de novo nucleotide biosynthesis	
Activate PARP	Cause ATP depletion	
Cause Ca^2+^ ions release from the ER lumen	Induce ER stress-mediated apoptosis	Lead to cancer cell death
Oxidize cardiolipin	Trigger mitochondria-mediated apoptosis	
Activate ASK-1		
Trigger cancer cell DNA damage	Activate P53-medicated apoptosis	
Reverses the R273H mutant P53 protein		
Reactivate the R175H mutant P53 protein		

**Table 2:** The promoting effects of ROS in cancer

Function	Result	Effect
Oxidize nucleobases	Form oxidative damage products	DNA mutation, subsequently promoting cancer formation
BRCA2 degradation	Inhibit BRCA2	
Oxidize hOGG1	Inhibit hOGG1 activity	
Stabilize HIF-1α	Interfere with T-cell development	Inhibit T cell function, finally promoting cancer development and metastasis.
Reduce CD3ζ chain expression	Block T cells differentiation and activation	
Phosphorylate ERK	T cells apoptosis	
Downregulate CD16ζ expression	Inhibit NK cells cytotoxicity	Inhibit NK cell function, subsequently promoting tumor growth and metastasis
Attenuate eIF-2B phosphorylation	Suppress ADCC of NK cell	
Inactivate PTEN	Macrophage recruitment, M2 polarization of macrophage	Promote tumor growth, invasion and metastasis
Activate TNF-α/TNFR1		
Activate NF-κB	Lose cell-cell junction	Trigger EMT process in cancer cells, finally promoting cancer cell invasion and metastasis
Promote TGF-β expression		
Activate HIF-1α		
Cause KEAP1 degradation		
Activate PI3K/Akt		
Oxidize 14-3-3ζ	Cytoskeleton remodeling	
Induce Arp2/3 recruitment		
Inhibit protein tyrosine phosphatase		
Activate MAPK, AP-1 and NF-κB	ECM degradation	

## Abbreviations

ROS: reactive oxygen species
H_2_O_2_: hydrogen peroxide
ER: endoplasmic reticulum
NOX: nicotinamide adenine dinucleotide phosphate oxidase
DNA: deoxyribonucleic acid
KRAS: Kirsten rat sarcoma viral oncogene
NK: natural killer
EMT: epithelial-mesenchymal transition
Ras: rat sarcoma
G: guanine
8-oxo-dG: 7,8-dihydro-8-oxo-2´-deoxyguanosine
A: adenine
T: thymine
C: cytosine
2-OH-dA: 2-hydroxy-2'-deoxyadenosine
8-oxo-dA: 7,8-dihydro-8-oxo-2'-deoxyadenosine
BER: base excision repair
NER: nucleotide excision repair
HR: homologous recombination
hOGG 1: human 8-oxoguanine DNA N-glycosylase 1
VEGF: vascular endothelial growth factor
HIF-1 α: hypoxia-inducible factor 1 α
ERK: extracellular signal-regulated kinase
PARP: poly(ADP-ribose) polymerase 1
HCC: hepatocellular carcinoma
NKG2D: natural killer group 2 member D
IFN-γ: interferon-γ
CMML: chronic myelomonocytic leukemia
TAMs: tumor-associated macrophages
PI3K: phosphatidylinositide 3-kinase
Akt: protein kinase B
CSF-1: colony-stimulating factor-1
TNF-α: tumor necrosis factor
IEC: intestinal epithelial cells
E-cadherin: epithelial cadherin
ECM: extracellular matrix
NF-κB: nuclear factor-κB
TGF-β: transforming growth factor-β
SMAD: trimeric drosophila mothers against decapentaplegic protein
NRF2: nuclear factor erythroid 2-related factor 2
KEAP1: Kelch-like ECH-associated protein 1
NOTHC1: translocation-associated (Drosophila)
GSK-3β: glycogen synthase kinase 3β
F-actin: polymer fiber actin
G-actin: monomer spherical actin
SSH-1L: slingshot-1L
FAK: focal adhesion kinase
MMPs: matrix metalloproteinases
AP-1: activator protein-1
MAPK: mitogen-activated protein kinase
uPA: urokinase plasminogen activator
uPAR: uPA receptor
HuR: Hu antigen R
ARE: AU-rich element
EGF: epidermal growth factor
EGFR: EGF receptor
G1: gap 1
S: DNA synthesis
M: mitosis
CDK: cyclin-dependent kinase
JAK: Janus kinase
STAT: signal transducers and activators of transcription
cdc25: cell division cycle 25
CHOP: C/EBP homologous protein
JNK: c-Jun NH(2)-terminal kinase
Cyt c: cytochrome c
MOMP: mitochondrial outer membrane permeabilization
BAX: Bcl-2-associated X protein
BAK: Bcl-2-antagonist killer
ATM: ataxia telangiectasia mutated
ATR: ataxia telangiectasia and Rad3-related
HO-1: heme oxygenase-1
PRDX1: peroxiredoxin 1
AMPK: 5′-adenosine monophosphate-activated protein kinase
KHK-A: fructokinase A
α-HB: α-hydroxybutyrate
KLF14: Krüppel-like factor 14
CBP: CREB-binding protein
Chk2: checkpoint kinase 2
PKM2: pyruvate kinase M2
GSH: glutathione
LIPG: endothelial lipase
RT: radiotherapy
PDT: photodynamic therapy
NPs: nanoparticles
DOX: doxorubicin
GG: galangin
IO NPs: iron oxide NPs
CONPs: cerium oxide NPs
LAHP: linoleic acid hydroperoxide
^1^O_2_: singlet oxygen
Hb: hemoglobin
